# Metabolomics and databases driven approach of identification of phytochemicals from seed of *Salvia schimperi* using high-performance liquid chromatography tandem mass spectrometry

**DOI:** 10.1371/journal.pone.0335929

**Published:** 2025-11-03

**Authors:** M. Azene, O. O. Olaokun, B.C. Iweriebor, N.M. Mkolo, C.L. Obi, W. Shibeshi, S. Assefa, Z. Abebe, A. Habtamu, K. Baye

**Affiliations:** 1 Center for Food Science, College of Natural and Computational Sciences, Addis Ababa University, Addis Ababa, Ethiopia; 2 School of Science and Technology, Department of Biology and Environmental Science, Sefako Makgatho Health Sciences University, Pretoria, South Africa; 3 Department of Pharmacology and Clinical Pharmacy, School of Pharmacy, College of Health Sciences, Addis Ababa University, Addis Ababa, Ethiopia; 4 Department of Pediatrics and Child Health, College of Health Sciences, Addis Ababa University, Addis Ababa, Ethiopia; Federal University of Espirito Santo, BRAZIL

## Abstract

*Salvia schimperi* is widely used in Ethiopian folk medicine, particularly its raw and roasted seeds for treating ailments such as diarrhea. While numerous reports on its traditional uses and biological activities exist, limited chemical datasets are available on this plant. This study aimed to annotate and identify the phytochemical constituents in *S. schimperi* seed extracts. The ground samples of *S. schimperi* seeds (raw and roasted) were extracted with 80% methanol prior to metabolomic analysis using LCMS. Data processing and compound identification were conducted using MZmine, SIRIUS and XCMS platforms. Multivariate statistical analyses and biological targets prediction were carried out via XCMS, MetaboAnalyst, BindingDB and Therapeutic Target Databases. Annotation by SIRIUS based on ZODIAC, SIRIUS and confidence scores criteria, validated 99 of the 689 identified compounds. Among the compounds annotated by METLIN in XCMS, 105 were predicted to be of significant value based on multivariate analysis of MetaboAnalyst. Compounds annotated comprised of primary and secondary metabolites. Two alkaloid compounds (actinonin and indole acrylate) were identified as significant from SIRIUS and METLIN/MetaboAnalyst, with actinonin described as a potent antibacterial metabolite. To our knowledge, this study represents the first metabolomic fingerprinting of *S. schimperi* seed extracts, revealing diverse bioactive phytochemicals with nutritional and therapeutic potential. The consistent identification of actinonin suggests promising antibacterial applications. Roasting significantly alters the metabolite profile, reducing certain nutritional components such as isoleucine. These findings underline the importance of processing methods in determining phytochemical composition and bioactivity. Further research would explore the toxicity and potential functional food applications.

## Introduction

In many developing countries, medicinal plants are essential as they offer a viable alternative in primary health care systems. The use of herbal medicine in these regions is motivated by various factors, including the perceived lower side effects and cost of phytomedicines compared to modern synthetic drugs, as well as the effectiveness of some plant remedies [1]. One of such medicinal plants is *Salvia schimperi*, commonly known as Schimper’s sage. This is a perennial shrub belonging to the Lamiaceae family, which includes about 900 species worldwide. Many species and varieties of the genus *Salvia* are cultivated for their aromatic nature and are used as flavoring and food condiments, as well as in cosmetics, perfumes, and medicine [2,3].

*S. schimperi* has various medicinal properties utilized in traditional medicine. Phytochemical analysis of the extracts revealed the presence of polyphenolic compounds such as flavonoids and tannins [4]. Research has reported on the chemical constituents of the essential oils of the leaves and flowers of *S. schimperi* [3] and the antinociceptive effects of the essential oil of its leaves [5]. The essential oils of the flowers and leaves contain compounds such as linalool, linalyl acetate, and α-terpineol [3], which contribute to their effectiveness against a range of pathogens, including Gram-positive and Gram-negative bacteria, as well as certain fungal strains [6]. The oils also demonstrate free-radical scavenging potential [6]. Additionally, compounds such as caryophyllene, bisabolol, and farnesene found in the essential oils of *S. schimperi* have been linked to its promising antidiabetic potential [7]. Despite the reports on its traditional uses and biological activities, limited chemical datasets are available for this plant. In particular there is a dearth of information on the phytochemicals and biological activities of the seed extracts.

Phytochemicals, or plant secondary metabolites, are essential for plants’ adaptation to environmental changes and interactions with other organisms [8–10]. These compounds are a sustainable source of lead compounds for drug discovery [11,12]. Characterizing phytochemicals is fundamental to the study of plant extracts, and advancements in technology over the past two decades have greatly enhanced this process [13,14]. Liquid chromatography coupled with mass spectrometry (LC-MS) has emerged as a preferred method due to its high throughput, soft ionization, and extensive coverage of phytochemicals [15]. Its versatility and sensitivity make LC-MS ideal for analyzing a wide variety of semi-polar compounds, including key secondary metabolites [13]. Tandem mass spectrometry (MS/MS) is particularly valuable as it provides detailed structural insights by capturing both precursor and fragment ion information, which are crucial for annotating, identifying, and dereplicating phytochemicals [16].

Phytochemical metabolomics analysis via LC-MS can be divided into untargeted and targeted approaches. The untargeted approach comprehensively analyzes all measurable chemicals, including unknowns, while the targeted approach focuses on specific predefined chemical groups [13]. LC-MS, a predominant tool for rapid characterization of these metabolites in herbal medicines, offers high resolution and sensitivity with multistage fragmentation data for detailed structural information [8,13]. Metabolites annotation tools such as MZmine, XCMS and SIRIUS aid in these analyses, facilitating the structural characterization of phytochemical mixtures and accelerating the discovery of novel active compounds [17,18].

Notably, annotating phytochemicals from untargeted mass spectrometry (MS) data often relies on spectral matching against limited reference databases [8]. Molecular networking (MN) has emerged as an effective approach for organizing complex MS/MS spectra by grouping structurally related compounds based on spectral similarity [19]. However, despite its advantages, the dereplication capabilities of MN are constrained by the availability of reference spectra and its limited ability to deeply annotate structurally similar compounds [20]. To address these limitations, several *in silico* tools such as MetFrag, MetFusion, ISIS, CFM-ID, and MAGMa have been developed [8]. Additionally, CSI:FingerID, integrated into the SIRIUS platform, uses machine learning to compute fragmentation trees and match experimental spectra with user-defined structure databases, thereby enhancing metabolite annotation coverage [21,22].

Therefore, this study aimed to annotate and identify the phytochemical constituents in *S. schimperi* seed extracts. The ground samples of *S. schimperi* seeds (both raw and roasted) were extracted with 80% methanol and subjected to untargeted High-Performance Liquid Chromatography Tandem Mass Spectrometry analysis. Data preprocessing and compounds annotations were conducted using web-based tools.

## Materials and methods

### Sample material

A sample of *S. schimperi* seeds were collected from Menz Mama Midir district, North Shewa Zone, Ethiopia between January 15 and February 14, 2022, identified and recorded as a voucher number of MA001 at National Herbarium, Addis Ababa University, College of Natural and Computational Sciences. The voucher specimen (voucher number MA001) was deposited at the National Herbarium, Addis Ababa University. The samples were collected, manually cleaned, dried and packed in hermetic plastic vessels, and stored at 5ºC until analysis. Roasting was performed at 185ºC for 15 minutes [23] using an electrical drying oven as communities living in Menz Mama Midir use the seed in roasted and raw forms.

### Instruments and reagents

Ultimate 3000 LC combined with Q Exactive MS (Thermo), Temp functional Centrifugation (Eppendorf), ACQUITY UPLC HSS T3 (100 × 2.1 mm × 1.8 μm), Acetonitrile (Merck), Methanol (Merck), Formic acid (Merck) and DL-o-Chlorophenylalanine (Merck) was used.

### Sample preparation

Each sample of *S. schimperi* seed (raw and roasted seeds) was crushed to fine powder in a 5-mL homogenizing tube, at 30 Hz with the aid of four 5-mm metal balls on a MM 400 mill mixer. Then 50 mg of each sample was precisely weighed into a tube and 800 μL of 80% methanol was added. Thereafter samples were vortexed for 30 s, followed by sonication for 30 min, 4°C. All samples were kept at −20°C for 1 h and centrifuged at 12000 rpm and 4°C for 15 min. Finally, 200 μL of supernatant and 5 μL of DL-o-Chlorophenylalanine (140 μg/mL) were transferred to the vial for LC-MS analysis.

### Plant samples data acquisition using UPLC-MS/MS

The analysis of secondary metabolites was performed using an Ultimate 3000 LC system (Thermo) coupled to a Q Exactive Orbitrap mass spectrometer (Thermo Fisher Scientific) equipped with an electrospray ionization (ESI) source. Separation was achieved on an ACQUITY UPLC HSS T3 column (100 × 2.1 mm, 1.8 μm). The mobile phases consisted of solvent A (0.05% formic acid in water) and solvent B (acetonitrile). The elution gradient program was as follows: (0–1 min, 95% A; 1–12 min, linear gradient from 95% A to 5% A; 12–13.5 min, 5% A (isocratic); 13.5–13.6 min, return from 5% A to 95% A; 13.6–30 min, re-equilibration at 95% A). The flow rate was 0.3 mL·min ⁻ ¹, the column temperature was maintained at 40 °C, and the autosampler was set at 4 °C. Mass spectrometric data were acquired in positive ionization mode under the following conditions: ESI^+^: heater temp 300 °C, spray voltage 3.5 kV, capillary temperature 350 °C, sheath gas flow rate 45 arb units, S-Lens RF level at 30%, auxiliary gas flow rate 15 arb units, resolution 70,000 (at m/z 200), and scan range m/z 100–1200.

### Data analysis software

Sample data acquired in centroid mode for the untargeted metabolomics analyses of methanol extracts from untreated (raw) and treated (roasted) seeds of *S. schimperi* was processed using the following software:

### MZmine

MZmine software (v3.7.0, Windows version) (http://mzmine.github.io/, downloaded on October 24, 2023) [24] was utilized to preprocess the raw LC-MS spectra data. Firstly, the raw data was imported into the platform, and data processing was performed, including mass detection (centroid), chromatogram resolving, deisotoping, peak alignment, and filling in missing peak data. The noise level parameters were set for MS1 (2.0E3) and MS2 (3.0E2) before executing the ADAP chromatogram builder with a scan-to-scan accuracy set at 0.005 Da or 10 ppm, while keeping other parameters at default values. Finally, the resulting MS1 feature data was exported and save in Excel (.csv) file while the MS2 feature data was exported and saved in an MGF file in SIRIUS/CSI:FingerID format.

### SIRIUS

SIRIUS software (v5.0, Windows version) (https://bio.informatik.uni-jena.de/software/sirius/, downloaded on November 6, 2023) [22] was used for the MS2 for compound annotation after the MGF file from the MZmine was imported into the software. Then for formula prediction, the program was set to search the entire database while enabling ZODIAC, CSI:FingerID, and CANOPUS other parameters were left at their default values. The “compute all” tab was selected to run the annotation by molecular formula prediction and structure elucidation step. In the Sirius overview tab where the results were displayed, the compound with the highest Sirius score was selected as the potential candidate. In addition to this, the “export summarize” option was enabled to facilitate the export of MS2 annotation search results of recommended compounds. The identified compounds were generated according to their confidence scores [25].

### XCMS

The analysis of LC-MS spectra data of untreated and treated samples was facilitated using the XCMS online [26] and on R package [27]. For the XCMS online (https://xcmsonline.scripps.edu/) spectra data was imported onto the platform using the pairwise drop-down on create new job. Then HPLC/single quad parameter was selected prior to running the program. For the XCMS (v3.20.0, an R package) the command line utilized to process spectra data. XCMS was used for feature extraction, feature alignment and data analysis, Annotation of compounds was enabled by the METLIN on XCMS online [28–31]. The resulting Excel csv files showcasing the total aligned peak intensity features of both XCMS analyses were compared. The XCMS online was selected for comparison of the untreated and treated samples by focusing on the feature extraction capability.

### BindingDB and therapeutic target database for biological targets prediction

The prediction of biological target of compounds was conducted using the BindingDB and Therapeutic Target Database. The exported recommended compounds from the Sirius MS2 search were further screened using their Simplified Molecular Input Line Entry System (SMILES) numbers. Each compound’s canonical SMILES was imported into the BindingDB database web tool (open-source license) to identify potential protein targets by clicking the “Find My Compound’s Target” button under the “Special tools” section. The biological targets in BindingDB were filtered for 85% similarity to existing ligand molecules [32]. Additionally, drug similarity was analyzed using the Therapeutic Target Database (TTD) web tool (open source license) [33].

### Statistical analyses

The resulting peak intensity features of XCMS online was subjected to a statistical analysis including using the MetaboAnalyst 5.0 [34]. The processed data was exported as CSV files containing information of the detected peaks, retention time and m/z values, to MetaboAnalyst 5.0. Data were normalized by sum, auto scaled and analyzed using principal component analysis (PCA) and orthogonal partial least-squares discriminant analysis (OPLS-DA). For the PCA, five components were analyzed to explain the variance between untreated and treated extracts. Then the OPLS-DA was used to further assess the significant difference between secondary metabolites extracted from the untreated and treated extracts. Multivariate data analyses tools were used to reveal signature metabolites that gratify the basis of *p*-value < 0.05, false discovery rate (FDR) corrected p-values (q-values) < 0.05, fold change (FC) > 2.0, and variable importance in projection (VIP) > 1.2. Cross-validation (CV) was performed using a tenfold CV method indicating the accuracy, Q^2^ and R^2^ values. A permutation test was also done to validate the model with the permutation p-value of p < 0.01.

## Result and discussion

This study represents one of the first attempts to investigate and describe the metabolomic profile of *S. schimperi* seed, specifically comparing roasted (ROS) (treated) and unroasted (RAW) (untreated) seed methanol extracts. The primary goal was to identify metabolomic differences, focusing on secondary metabolites such as polyphenols (e.g., flavonoids), terpenoids, alkaloids, and other compounds, which may serve as bio-fingerprints and with possible antibacterial potential. An untargeted metabolomics analysis was conducted using LC-MS/MS (liquid chromatography–tandem mass spectrometry). This high-resolution MS data acquisition method allowed for the separation and possible detection of numerous phytochemicals, identified by their mass-to-charge ratio (m/z) and retention time (RT).

### Data processing on MZmine and phytochemical annotation using SIRIUS 5.0

**Fig 1:**
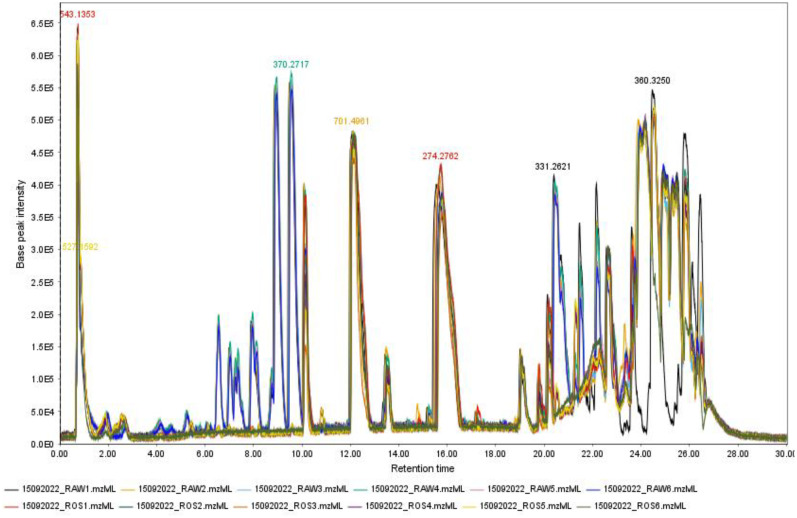
Total ion chromatography of *S. schimperi* seed methanol extracts (treated (ROS) and untreated.

The LC-MS spectra obtained from the methanolic extracts of both treated and untreated *S. schimperi* seeds were subjected to peak detection and integration, resulting in the generation of 2D matrices. These matrices included variable indices (paired m/z-retention time), sample identifiers (observations), and peak areas. Several software packages are available for the processing of metabolomic data, but their performance can vary significantly, potentially leading to different outcomes. In this study, we evaluated the quality of the processed data matrices obtained using two freely available tools, XCMS and MZmine, by comparing them with the raw data. MZmine, XCMS Online, and the XCMS R package identified 774, 2,756, and 2,530 m/z features, respectively (Table 1). MZmine detected the fewest variables/peaks, possibly because some extracted noise peaks were removed directly through visual inspection. The total ion chromatogram (TIC) of *S. schimperi* extracts (Fig 1), obtained through a 30-minute gradient elution, revealed MS1 features ranging from m/z 100.0223 to 999.2317 and the maximum TIC intensity reached 2.9 × 10⁶.

**Table 1:** Data processing results summary.

Software	Initial detected features	Total aligned features
MZmine	16,527	774
XCMS Online	16,527	2756
XCMS Online on R	16,527	2530

SIRIUS platform computes candidate molecular formulas (MFs) by matching the experimental MS1 spectra against predicted isotopic patterns and assessing how well the fragmentation spectra align with candidate MFs through fragmentation trees. To enhance high degree annotation, SIRIUS integrates advanced algorithms such as ZODIAC for enhanced MF prediction, CSI:FingerID for putative structural annotation, and COSMIC for confidence scores, alongside CANOPUS for chemical class annotation [13]. For this study, the spectra features obtained from MZmine were imported into SIRIUS 5.0. Key attributes such as retention time, ion mass, adduct, confidence ranking and COSMIC confidence score were generated. Candidate structures for the compounds of *S. schimperi* were identified on SIRIUS platform by searching the top CSI:Finger ID hits across various databases, including Natural Products, COCONUT, and CHEBI. A confidence score (value between 0 and 1) given to each candidate predicted the probability of correct structural identification. The results generated were then manually curated, and the fingerprint vectors of the top candidates were exported for further analysis. From this workflow, a total of 689 compounds were identified in the extracts (S1 File in S1 Data) This comprised both primary and secondary metabolites, including fatty acids, carbohydrates, Shikimates and phenylpropanoids, alkaloids, terpenoids and polyamines. To ensure reliability, only candidates that passed the selection criteria thresholds were retained. Specifically, compounds with a COSMIC confidence score > 0.1 were included while no identification was made if the ZODIAC score was below 50% or if the SIRIUS score was below 50% without a corresponding ZODIAC score [35–38]. Based on this selection, only 99 compounds were validated (Table 2), which were further categorized into 25 chemical classes (Table 3). In addition, the export summary function of SIRIUS, which is designed to automatically annotate and recommend compounds based on MS2 spectral data, generated annotations for ten compounds as shown in Table 4. However, two of these compounds; 2-[4-(4-methoxyphenyl)-2,2-dimethyloxan-4-yl]ethyl-[(4-methoxyphenyl)methyl]azanium, and 2-methyl-4,6-dinitro-5-(4-phenylpiperazin-1-yl)-1H-benzimidazole were absent in Table 2. This is because both compounds failed to satisfy the scoring thresholds set to ensure high-confidence identification. The complementary use of COSMIC and CANOPUS further strengthened the annotation process. COSMIC provided the confidence levels for molecular structure annotations for selected compounds, while CANOPUS offered broad classification of all measured compounds into chemical classes. This integrated approach yielded a comprehensive insight into the phytochemical profile *S. schimperi*. This approach has been successfully employed in previous studies, including the detailed chemical characterization of the Pingxiao capsule [39]. In the present study, it proved equally effective, revealing that the identified compounds were predominantly alkaloids, fatty acids, amino acids, and peptides.

**Table 2:** Compounds in extracts of *S. schimperi* based on confidence, Zodiac and Sirius scores in SIRIUS 5.0 software.

Compound Name	Confidence Score	Zodiac Score	Sirius Score	Molecular Formula	Adduct	xLogP	Ion Mass (m/z)	Retention Time (min)
Dilinoleoyl-PE	0.160	1	10030.6	C₄₁H₇₄NO₈P	[M ⁺ Na]⁺	9.2	762.5	23.3
Phophatidylethanolamine(18:2n6/18:3n3)	0.155	1	9034.4	C₄₁H₇₂NO₈P	[M ⁺ Na]⁺	8.5	760.5	26.1
Lysophosphatidylethanolamine(18:2/0:0)	0.496	1	9012.4	C₂₃H₄₄NO₇P	[M ⁺ Na]⁺	1.8	500.3	21.2
Dilinolenoyl lecithin	0.293	1	8997.7	C₄₄H₇₆NO₈P	[M ⁺ Na]⁺	11.1	800.5	25.8
1-16:0-2-18:3-phosphatidylcholine	0.245	1	8992.2	C₄₂H₇₈NO₈P	[M ⁺ Na]⁺	12.3	778.5	23.8
Brain PC	0.284	1	8006.3	C₄₂H₈₂NO₈P	[M ⁺ Na]⁺	13.7	782.6	26.4
Dlpc lipid	0.334	1	8004.2	C₄₄H₈₀NO₈P	[M ⁺ Na]⁺	12.5	804.6	23.9
Phosphatidylcholine(20:3n6/14:0)	0.316	1	8000.9	C₄₂H₇₈NO₈P	[M ⁺ Na]⁺	12.3	778.5	23.8
1-linoleoyl-2-palmitoyl-sn-glycero-3-phosphocholine	0.269	1	7999.1	C₄₂H₈₀NO₈P	[M ⁺ Na]⁺	13.0	780.6	24.9
1,2-docpc	0.196	1	7980.3	C₄₄H₈₄NO₈P	[M ⁺ Na]⁺	13.8	808.6	23.2
Phophatidylethanolamine(18:3n3/18:2n6)	0.185	1	7022.3	C₄₁H₇₂NO₈P	[M ⁺ H]⁺	8.5	738.5	26.1
N-[(2S,3R,4E,8Z)-3-hydroxy-1-[(2R,3R,4S,5S,6R)-3,4,5-trihydroxy-6-(hydroxymethyl)oxan-2-yl]oxyoctadeca-4,8-dien-2-yl]hexadecanamide	0.433	1	7021.6	C₄₀H₇₅NO₈	[M ⁺ O ⁺ H]⁺	10.3	714.6	25.4
PC(18:1omega9/16:0)	0.284	1	7018.2	C₄₂H₈₂NO₈P	[M ⁺ Na]⁺	13.7	782.6	26.4
Phosphatidylcholine(20:1/14:0)	0.285	1	7011.3	C₄₂H₈₂NO₈P	[M ⁺ Na]⁺	13.7	782.6	26.4
Phosphatidylcholine(18:3n6/18:2n6)	0.159	1	7010.2	C₄₄H₇₈NO₈P	[M ⁺ Na]⁺	11.8	802.5	26.5
Phophatidylethanolamine(14:0/20:2)	0.199	1	7006.1	C₃₉H₇₄NO₈P	[M ⁺ H]⁺	9.7	716.5	24.4
PC(18:1omega9/18:2omega6)	0.259	1	7005.1	C₄₄H₈₂NO₈P	[M ⁺ Na]⁺	13.2	806.6	25.3
1-myristoyl-2-stearoyl-sn-glycero-3-phosphoethanolamine	0.164	1	6998.8	C₃₇H₇₄NO₈P	[M ⁺ Na]⁺	10.3	714.5	23.0
1-a-linolenoyl-2-stearoyl-sn-glycero-3-phosphoethanolamine	0.165	1	6995.6	C₄₁H₇₆NO₈P	[M ⁺ Na]⁺	10.1	764.5	24.8
Phosphatidylcholine(16:1/20:1)	0.257	1	6977.4	C₄₄H₈₄NO₈P	[M ⁺ Na]⁺	13.8	808.6	23.3
1-plpea	0.184	1	6974.3	C₃₉H₇₄NO₈P	[M ⁺ Na]⁺	9.7	738.5	24.4
1-16:0-lysopc	0.672	1	6037.3	C₂₄H₅₀NO₇P	[M ⁺ H]⁺	5.6	496.3	21.8
1-18:0-lysopc	0.444	1	6035.9	C₂₆H₅₄NO₇P	[M ⁺ H]⁺	6.7	524.4	23.0
2-18:2(9Z,12Z)-lysopc	0.595	1	6033.2	C₂₆H₅₀NO₇P	[M ⁺ H]⁺	5.1	520.3	20.9
1-homo-g-linolenoyl-2-palmitoyl-sn-glycero-3-phosphoethanolamine	0.172	1	6024.4	C₄₁H₇₆NO₈P	[M ⁺ H]⁺	10.1	742.5	24.8
1-18:2-lysophosphatidylcholine	0.589	1	6018.9	C₂₆H₅₀NO₇P	[M ⁺ Na]⁺	5.1	542.3	21.3
Phosphatidylcholine(18:3n3/18:2n6)	0.200	1	6017.0	C₄₄H₇₈NO₈P	[M ⁺ Na]⁺	11.8	802.5	26.5
1-16:0-lysope	0.606	1	6016.4	C₂₁H₄₄NO₇P	[M ⁺ Na]⁺	2.4	476.3	21.7
1-homo-g-linolenoyl-2-palmitoyl-sn-glycero-3-phosphocholine	0.322	1	6007.9	C₄₄H₈₂NO₈P	[M ⁺ Na]⁺	13.4	806.6	25.3
1-16:0-2-18:3-phosphatidylethanolamine	0.206	1	6005.5	C₃₉H₇₂NO₈P	[M ⁺ H]⁺	9.0	714.5	22.9
Phophatidylethanolamine(16:1/20:2)	0.153	1	6004.5	C₄₁H₇₆NO₈P	[M ⁺ H]⁺	9.9	742.5	24.8
1-(1Z-hexadecenyl)-2-(9Z-nonadecenoyl)-glycero-3-phosphoserine	0.208	1	6002.6	C₄₁H₇₈NO₉P	[M ⁻ H₂O ⁺ H]⁺	11.1	742.5	24.8
Phophatidylethanolamine(20:2/16:1)	0.154	1	6001.2	C₄₁H₇₆NO₈P	[M ⁺ H]⁺	9.9	742.5	24.7
1-linoleoyl-2-vaccenoyl-sn-glycero-3-phosphoethanolamine	0.154	1	6000.8	C₄₁H₇₆NO₈P	[M ⁺ H]⁺	9.9	742.5	24.8
1-eicosadienoyl-2-palmitoyl-sn-glycero-3-phosphocholine	0.265	1	5996.7	C₄₄H₈₄NO₈P	[M ⁺ Na]⁺	14.1	808.6	25.3
1-palmitoyl-2-eicosadienoyl-sn-glycero-3-phosphocholine	0.267	1	5996.1	C₄₄H₈₄NO₈P	[M ⁺ Na]⁺	14.1	808.6	25.3
1-a-linolenoyl-2-palmitoyl-sn-glycero-3-phosphoethanolamine	0.208	1	5995.7	C₃₉H₇₂NO₈P	[M ⁺ H]⁺	9.0	714.5	23.0
1-18:0-2-18:3-phosphatidylethanolamine	0.167	1	5990.2	C₄₁H₇₆NO₈P	[M ⁺ Na]⁺	10.1	764.5	24.8
Phosphatidylcholine(14:0/22:2)	0.272	1	5989.6	C₄₄H₈₄NO₈P	[M ⁺ Na]⁺	14.1	808.6	24.8
Phosphatidylcholine(18:0/18:2w6)	0.276	1	5971.7	C₄₄H₈₄NO₈P	[M ⁺ Na]⁺	14.1	808.6	24.8
1-18:3-2-18:3-phosphatidylethanolamine	0.328	1	5021.8	C₄₁H₇₀NO₈P	[M ⁺ H]⁺	7.8	736.5	25.6
1-vaccenoyl-2-linoleoyl-sn-glycero-3-phosphoethanolamine	0.152	1	5011.1	C₄₁H₇₆NO₈P	[M ⁺ H]⁺	9.9	742.5	24.8
1-18:3-lysopc	0.495	1	5010.1	C₂₆H₄₈NO₇P	[M ⁺ Na]⁺	4.4	540.3	20.5
1-18:1-lysope	0.312	1	5006.7	C₂₃H₄₆NO₇P	[M ⁺ Na]⁺	2.5	502.3	22.0
Phophatidylethanolamine(20:3n6/14:0)	0.113	1	5002.2	C₃₉H₇₂NO₈P	[M ⁺ H]⁺	9.0	714.5	23.0
Lysolecithin	0.306	1	5001.7	C₂₆H₅₂NO₇P	[M ⁺ Na]⁺	5.8	544.3	22.1
C16-PAF acether	0.390	1	4995.6	C₂₆H₅₄NO₇P	[M ⁺ Na]⁺	6.6	546.4	23.0
Phosphatidylcholine(14:0/20:2)	0.260	1	4988.4	C₄₂H₈₀NO₈P	[M ⁺ Na]⁺	13.0	780.5	25.8
2-Linoleoyl-1-palmitoyl-rac-glycero-3-phosphocholine-(trimethyl-d9)	0.181	1	4987.0	C₄₃H₈₂NO₈P	[M ⁺ Na]⁺	13.5	794.6	25.7
Butenoyl PAF	0.403	1	4007.2	C₂₈H₅₆NO₇P	[M ⁺ H]⁺	7.4	550.4	23.2
Lysophosphatidylcholine(20:0)	0.597	1	4006.2	C₂₈H₅₈NO₇P	[M ⁺ H]⁺	7.8	552.4	24.0
Phosphatidylglycerol(18:3n6/16:1n7)	0.277	1	3997.4	C₄₀H₇₁O₁₀P	[M ⁺ Na]⁺	10.0	765.5	23.7
Phosphatidylcholine(20:2/14:0)	0.262	1	3971.8	C₄₂H₈₀NO₈P	[M ⁺ Na]⁺	13.0	780.5	25.8
PE(16:0/20:4omega6)	0.152	1	3006.4	C₄₁H₇₄NO₈P	[M ⁺ H]⁺	9.4	740.5	24.3
1-nonadecanoyl-sn-glycero-3-phosphocholine	0.362	1	3004.9	C₂₇H₅₆NO₇P	[M ⁺ H]⁺	7.3	538.4	23.4
1-stearoyl-phosphatidylethenolamine	0.334	1	3003.7	C₂₃H₄₈NO₇P	[M ⁺ H]⁺	3.4	482.3	22.9
Diacylglycerol(18:3n6/18:3n3)	0.137	1	3003.2	C₃₉H₆₄O₅	[M ⁺ Na]⁺	11.6	635.5	24.3
1-heneicosanoyl-glycero-3-phosphate	0.418	1	3000.8	C₂₄H₄₉O₇P	[M ⁺ C₂H₃N ⁺ Na]⁺	7.8	544.3	21.8
Glyceryl dilinoleate	0.176	1	2992.6	C₃₉H₆₈O₅	[M ⁺ Na]⁺	13.0	639.5	23.5
Diacylglycerol(18:2n6/18:3n3)	0.129	1	2991.1	C₃₉H₆₆O₅	[M ⁺ Na]⁺	12.3	637.5	25.7
Pristanoylglycine	0.151	1	2013.9	C₂₁H₄₁NO₃	[M ⁺ O ⁺ H]⁺	7.2	372.3	22.2
Diacylglycerol(18:2/16:0)	0.124	1	2013.4	C₃₇H₆₈O₅	[M ⁺ Na]⁺	13.5	615.5	25.6
2-[[2-acetyloxy-3-[(E)-hexadec-9-enoxy]propoxy]-hydroxyphosphoryl]oxyethyl-trimethylazanium	0.193	1	2003.8	C₂₆H₅₂NO₇P	[M ⁺ H]⁺	5.7	522.4	21.8
Phosphatidylcholine(20:3n6/15:0)	0.182	1	2001.5	C₄₃H₈₀NO₈P	[M ⁺ H]⁺	12.9	770.6	24.8
1-pentadecanoyl-glycerol	0.450	1	1017.8	C₁₈H₃₆O₄	[M ⁺ H₃N ⁺ H]⁺	5.1	334.3	16.3
1-butyl-3-[4-[2-hydroxy-3-(propan-2-ylamino)propoxy]phenyl]urea	0.221	1	134.8	C₁₇H₂₉N₃O₃	[M ⁺ H]⁺	2.4	324.2	7.0
Lopac-U-1508	0.184	1	128.5	C₂₂H₃₅NO₃	[M ⁺ Na]⁺	4.1	384.3	7.3
Erucylamide	0.208	1	113.8	C₂₂H₄₃NO	[M ⁺ H]⁺	8.8	338.3	24.5
N-pentadecylcyclohexanecarboxamide	0.236	1	110.1	C₂₂H₄₃NO	[M ⁺ H]⁺	8.8	338.3	24.5
2,2’‘-Oxybis(N,N-dioctylacetamide)	0.243	1	100.5	C₃₆H₇₂N₂O₃	[M ⁻ H₂O ⁺ H]⁺	12.8	563.6	22.6
3-[1-(3-cyclohexyl-3-hydroxypropyl)-3,4-dimethylpiperidin-4-yl]phenol	0.303	0.80	92.0	C₂₂H₃₅NO₂	[M ⁺ Na]⁺	5.2	368.3	8.7
1-monolinolenin	0.381	1	86.1	C₂₁H₃₆O₄	[M ⁺ H]⁺	5.3	353.3	21.5
Bmse010049	0.513	1	79.6	C₂₄H₂₆O₈	[M ⁺ Na]⁺	2.5	465.2	15.2
1,2-Bis(sulfonyl)-1-methylhydrazine	0.211	1	77.8	C₃H₁₀N₂O₄S₂	[M ⁺ H]⁺	−1.2	203.0	0.1
(?14(15)-eet ethanolamide	0.198	1	75.7	C₂₂H₃₇NO₃	[M ⁺ Na]⁺	4.4	386.3	7.3
Sclareol	0.151	1	75.2	C₂₀H₃₆O₂	[M ⁻ H₄O₂ ⁺ H]⁺	4.9	273.3	20.4
Alpha-LA	0.115	1	74.6	C₁₈H₃₀O₂	[M ⁺ H]⁺	5.9	279.2	22.3
Oprea1_159358	0.287	0.95	71.5	C₂₀H₁₈O₄	[M ⁺ H]⁺	2.9	323.1	15.2
Oleamide	0.357	1	67.2	C₁₈H₃₅NO	[M ⁺ H]⁺	6.6	282.3	22.6
3,5-ditert-butylbenzaldehyde	0.123	1	66.9	C₁₅H₂₂O	[M ⁺ H]⁺	4.7	219.2	16.6
Stigmastan-3,5-diene	0.171	1	66.7	C₂₉H₄₈	[M ⁺ H]⁺	10.6	397.4	25.4
Oprea1_749540	0.208	1	66.6	C₁₁H₉NO₂	[M ⁺ H₃N ⁺ H]⁺	2.6	205.1	5.3
Indol-acrylate	0.429	1	64.6	C₁₁H₉NO₂	[M ⁺ H₃N ⁺ H]⁺	1.8	205.1	5.3
1,2-Diacetyl-3-decanoylglycerol	0.523	0.99	64.4	C₁₇H₃₀O₆	[M ⁺ H]⁺	3.6	331.2	20.2
Naproxen impurity k	0.137	1	62.3	C₁₃H₁₄O₂	[M ⁺ H]⁺	2.9	203.1	17.7
(-)-Yatein	0.231	0.84	62.1	C₂₂H₂₄O₇	[M ⁻ H₂O ⁺ H]⁺	3.8	383.2	15.2
Aschantin	0.190	1	61.9	C₂₂H₂₄O₇	[M ⁻ H₂O ⁺ H]⁺	2.8	383.2	15.2
(?5(6)-eet ethanolamide	0.135	1	60.6	C₂₂H₃₇NO₃	[M ⁺ Na]⁺	4.5	386.3	7.9
ACMC-20mzcj	0.114	1	60.2	C₁₈H₃₀O₂	[M ⁺ H]⁺	5.9	279.2	22.3
Isomollupentin 7-O-glucoside-2’“‘-O-xyloside	0.296	0.66	56.1	C₃₁H₃₆O₁₈	[M ⁺ K]⁺	−3.1	735.2	0.9
1-butyl-5-methyl-3,7-dipropanoyl-3,7-diazabicyclo[3.3.1]nonan-9-one	0.317	1	55.7	C₁₈H₃₀N₂O₃	[M ⁺ H₃N ⁺ H]⁺	1.6	340.3	12.1
Melitose	0.121	0.99	55.6	C₁₈H₃₂O₁₆	[M ⁺ Na]⁺	−6.2	527.2	0.9
Selin CLA	0.183	1	54.8	C₁₈H₃₂O₂	[M ⁺ H]⁺	6.3	281.2	22.9
Oprea1_805463	0.533	0.99	54.5	C₂₄H₂₈O₄	[M ⁺ K]⁺	5.0	419.2	20.1
Linolate	0.177	1	54.3	C₁₈H₃₂O₂	[M ⁺ H]⁺	6.3	281.2	22.9
Linoleamide	0.230	1	52.8	C₁₈H₃₃NO	[M ⁺ H]⁺	5.9	280.3	21.8
2,2’‘-((2-(Dodecyloxy)ethyl)imino)bisethanol	0.424	1	51.2	C₁₈H₃₉NO₃	[M ⁺ H]⁺	4.3	318.3	15.8
Actinonin	0.489	0.91	50.2	C₁₉H₃₅N₃O₅	[M ⁺ H]⁺	1.5	386.3	7.9
[(2R,3S,4S,5R,6R)-3,4,5-trihydroxy-6-[2-[4-(3,4,5-trihydroxybenzoyl)oxyphenyl]ethoxy]oxan-2-yl]methyl 3,4,5-trihydroxybenzoate	0.220	0.88	50.1	C₂₈H₂₈O₁₅	[M ⁺ Na]⁺	0.9	627.1	12.0

**Table 3:** Characteristics of class of the compounds identified by SIRIUS software.

Precursor formula	NPC# Pathway	Pathway probability	NPC# Class	Class probability
C₃H₁₀N₂O₄S₂	Amino acids and peptides	0.91	Amino acids	0.98
C₁₅H₂₂O	Terpenoids	0.97	Bisabolane sesquiterpenoids	0.52
C₁₅H₁₆N₄	Alkaloids	0.97	Carboline alkaloids	0.38
C₂₈H₂₈O₁₅	Shikimates and phenylpropanoids	0.99	Cinnamic acids and derivatives	0.85
C₃₇H₆₈O₅	Fatty acids	1.00	Diacylglycerols	1.00
C₂₂H₂₂O₆	Shikimates and phenylpropanoids	1.00	Dibenzylbutyrolactone lignans	0.85
C₁₈H₃₂O₁₆	Carbohydrates	1.00	Disaccharides	1.00
C₂₂H₂₂O₆	Shikimates and phenylpropanoids	1.00	Furofuranoid lignans	0.98
C₂₈H₅₈NO₇P	Fatty acids	1.00	Glycerophosphocholines	1.00
C₄₁H₇₆NO₈P	Fatty acids	1.00	Glycerophosphoethanolamines	1.00
C₄₀H₇₁O₁₀P	Fatty acids	1.00	Glycerophosphoglycerols	1.00
C₂₀H₃₂	Terpenoids	0.99	Labdane diterpenoids	1.00
C₁₇H₃₀O₆	Fatty acids	0.84	Macrolide lactones	0.31
C₂₁H₃₆O₄	Fatty acids	1.00	Monoacylglycerols	0.99
C₃₆H₇₀N₂O₂	Fatty acids	0.97	N-acyl amines	0.86
C₁₈H₃₉NO₃	Fatty acids	0.98	N-acyl ethanolamines (endocannabinoids)	0.81
C₄₀H₇₅NO₉	Fatty acids	1.00	Neutral glycosphingolipids	1.00
C₂₀H₁₈O₄	Shikimates and phenylpropanoids	0.77	Phenanthrenes	0.20
C₁₇H₂₉N₃O₃	Alkaloids	0.75	Phenylalanine-derived alkaloids	0.19
C₂₄H₂₈O₄	Terpenoids	0.91	Pregnane steroids	0.48
C₂₂H₄₃NO	Fatty acids	0.94	Primary amides	0.87
C₁₈H₃₃N₃O₃	Amino acids and peptides	0.70	Quinolizidine alkaloids	0.27
C₁₁H₉NO₂	Alkaloids	0.97	Simple indole alkaloids	0.81
C₁₉H₃₅N₃O₅	Amino acids and peptides	0.84	Tripeptides	0.41
C₁₈H₃₂O₂	Fatty acids	0.76	Unsaturated fatty acids	0.95

**Table 4:** Compounds identified using the MS2 spectra results of “export summarized” option enabled by SIRIUS 5.0 software.

Seq No	Compound name	NPC# pathway	NPC# pathway probability	Isotope Score	Tree Score	Mass Error Fragment Peaks (ppm)	Ion Mass	RT (s)
1	1-butyl-3-[4-[2-hydroxy-3-(propan-2-ylamino)propoxy]phenyl]urea	Alkaloids	0.77	3.20	68.62	1.805	324.23	420.18
2	Pacitron	Amino acids & peptides	1.00	0.00	57.64	0.971	205.10	314.71
3	Indol-acrylate	Alkaloids	0.94	0.00	55.21	1.144	205.10	314.76
4	3-(2’‘-Methylpiperidino)propyl p-hexoxybenzoate	Alkaloids	0.68	3.21	31.45	4.141	384.25	439.16
5	2-methyl-4,6-dinitro-5-(4-phenylpiperazin-1-yl)-1h-benzimidazole	Alkaloids	0.83	4.36	21.30	2.158	400.17	367.16
6	Dodecanamide, N-[3-hydroxy-1-(hydroxymethyl)-3-phenylpropyl]-	Alkaloids	0.91	2.70	20.61	1.683	386.27	433.96
7	(?5(6)-EET Ethanolamide	Alkaloids	0.72	3.27	18.41	1.279	386.27	473.48
8	Actinonin	Alkaloids	0.54	3.28	18.39	1.279	386.27	473.18
9	2-[4-(4-methoxyphenyl)-2,2-dimethyloxan-4-yl]ethyl-[(4-methoxyphenyl)methyl]azanium	Alkaloids	0.55	0.00	14.17	0.234	384.25	440.62
10	(2S)-1-[(2S)-2-[[(2S,3S)-2-amino-3-methylpentanoyl]amino]-5-(diaminomethylideneamino)pentanoyl]pyrrolidine-2-carboxylic acid	Amino acids & peptides	0.79	0.00	5.01	6.353	385.25	440.78

### Data analysis and phytochemical annotation using XCMS and METLIN

**Fig 2:**
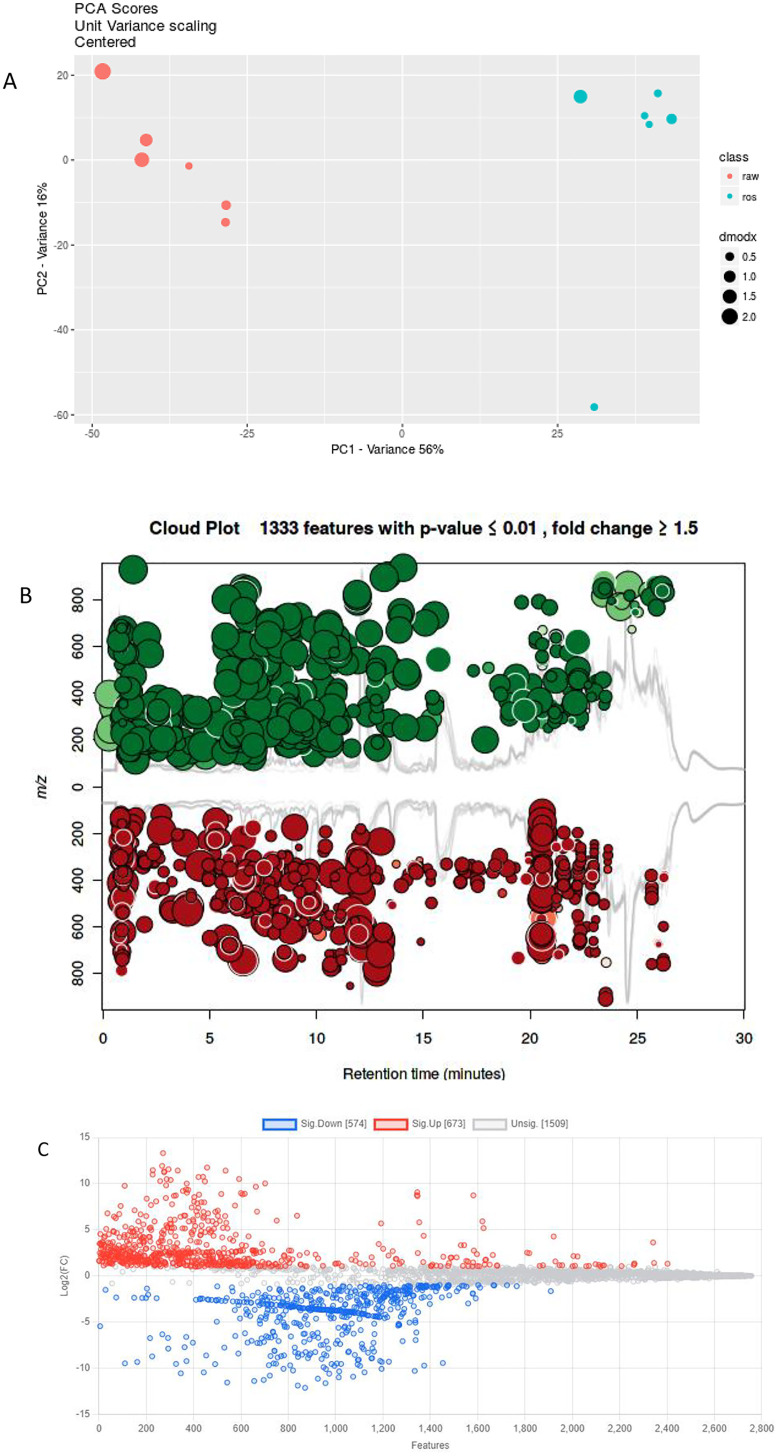
A. Visualization of features of *S. schimperi* seed extracts of raw (untreated) (RAW) and roasted (treated) (ROS) in Multivariate analysis PCA plot, illustrating PCA scores unit variance scaling of features based on XCMS online prediction. B. Visualization of features of *S. schimperi* seed extracts of raw (untreated) (RAW) and roasted (treated) (ROS) in Cloud plot of XCMS online, illustrating features significantly upregulated (color in green) and significantly downregulated (color in red). C. Visualization of features of *S. schimperi* seed extracts of raw (untreated) (RAW) and roasted (treated) (ROS) in Fold change on MetaboAnalyst webtool, illustrating sig. Up (significantly upregulated) (color in red), sig. Down (significantly downregulated) (color in blue), and unsig. (unsignificant) (color in grey).

The data processing done on XCMS Online and on R packages (Table 1) resulted in the difference of 226 m/z features, for which no clear explanation could be provided. XCMS Online, a cloud-based metabolomics informatics platform, has undergone significant improvements, including the incorporation of paired two-group comparisons, higher-order meta-analysis, and multiple group comparisons [40,41]. In addition, statistical tests have been introduced into it, and the interactive visualization tools have been improved and expanded to better deconvolve complex untargeted metabolomic datasets. These statistical tests were systematically performed following feature detection and profile alignment, providing a direct interface for visualization [40]. For the *S. schimperi* seed extracts, feature detection produced a mean value of 1995.5 ± 86.25 features in RAW (untreated) samples and 1952.81 ± 212.02 features in ROS (treated) samples (S2 File in S1 Data). The extracted features were then subjected to principal component analysis (PCA) to explore and visualize the metabolite variation between the two treatment groups. The PCA score plot revealed that the first principal component (PC1) accounted for 56.0% of the observed variation, while the second principal component (PC2) explained 16.0% (Fig 2A), clearly distinguishing between the RAW and ROS extracts. To further evaluate sample variability, the Distance to the Model of X-space (DModX) was calculated. The results indicated that three RAW samples and two ROS samples exhibited higher DModX values (Fig 2A), suggesting possible outliers with greater deviation from the PCA model. In PCA, a high DModX value reflects a point that departs significantly from the correlation structure captured by the model, thereby flagging it as a potential outlier.

Feature-level analysis in XCMS Online identified a total of 2756 features, of which 1081 were upregulated and 1675 were downregulated (S3 File in S1 Data). When filtered using significance thresholds of *p* ≤ 0.01 and fold change ≥ 1.5, a total of 1333 significant features were retained, comprising 589 upregulated and 744 downregulated features (Fig 2B). In contrast, analysis using MetaboAnalyst yielded 673 significantly upregulated features, 574 significantly downregulated features, and 1509 unsignificant features (Fig 2C). This difference underscores the variance in interpretation between the two analytical webtools.

The METLIN-guided in-source annotation (MISA) algorithm annotates m/z values of in-source fragments (ISFs) using experimental low-energy MS/MS spectra from the METLIN library, and is applied to the datasets generated by XCMS Online [40,41]. Through this process, a total of 2087 compounds were predicted, of which 451 were classified as unknowns. Among the known compounds, 105 metabolites were identified as significant based on the Significance Analysis of Metabolites (SAM) performed in MetaboAnalyst (S4 File S1 Data), with some of the compounds shown in Table 5.

**Table 5:** Significant compounds based on significant Analysis of Metabolites of *S. shimperi* extracts by METLIN on XCMS.

Seq No	Name of compound	Name	Fold of change	log2fold	T-stat	P-value	m/z	RT (mins)	Peak group	Up/down regulated
1	(2Z,4’Z)-2-(5-Methylthio-4-penten-2-ynylidene)-1,6-dioxaspiro[4.4]non-3-ene	M365T1	10686.4	−13.38	−28.47	1.00E-06	365.11	0.94	17	DOWN
2	3-[(2-Mercapto-1-methylpropyl)thio]-2-butanol	M217T1	5244.9	−12.36	−41.81	1.48E-07	217.07	1.02	35	DOWN
3	Arachidonoyl ethanolamide	M370T10	4269.6	−12.06	−36.54	2.89E-07	370.27	9.63	2	DOWN
4	Actinonin	M368T9	3411.6	−11.74	−36.06	3.09E-07	368.26	8.99	3	DOWN
5	Arachidonamide	M326T8	2903.5	−11.50	−36.77	2.80E-07	326.24	8.02	24	DOWN
6	Eicosa-5,8-dienoic acid (20:2 n-12)	M331T21	1215.5	−10.25	−41.38	1.55E-07	331.26	20.59	11	DOWN
7	Diprotin a	M324T7	868.6	9.76	11.16	0.0001	324.23	7.05	30	DOWN
8	Cyanidin 3-sambubioside	M614T1	819.1	−9.68	−37.91	2.38E-07	614.18	0.91	17	DOWN
9	Ara-G hydrate	M306T2	656.9	−9.36	−29.52	8.35E-07	306.08	1.95	65	DOWN
10	Sulfinpyrazone	M369T1	370.9	−8.54	−27.57	1.17E-06	369.11	0.99	35	DOWN
11	C17 sphinganine	M332T20	297.0	8.21	15.02	2.28E-05	332.25	20.47	37	DOWN
12	5-(1-methoxybutan-2-yl)isolongifol-5-ene	M273T21	287.3	8.17	26.85	1.14E-06	273.26	20.54	11	DOWN
13	Naphtho[1,2-b]phenazin-5(8H)-one, 6-chloro-8-ethyl	M323T5_1	233.8	−7.87	−26.86	1.33E-06	323.07	4.58	96	DOWN
14	Balanol; azepinostatin	M533T1	214.7	7.75	28.80	7.55E-07	533.16	0.89	17	DOWN
15	Dihomo-gamma-linolenoyl glycine	M386T8	137.7	−7.11	−59.97	2.02E-08	386.27	7.96	24	DOWN
16	Methoxyzotepine	M362T1	127.6	−7.00	−26.77	1.31E-06	362.10	0.93	17	DOWN
17	Delphinidin 3-caffeylglucoside	M627T12	101.8	−6.67	−56.43	3.02E-08	627.13	12.12	82	DOWN
18	Methyl arachidonyl fluorophosphonate	M393T21	99.0	6.63	24.77	8.38E-07	393.23	20.58	11	DOWN
19	N-hydroxy arachidonoyl amine	M342T6	93.5	−6.55	−30.16	7.47E-07	342.24	6.10	91	DOWN
20	2R-hydroperoxy-9Z,12Z,15Z-octadecatrienoic acid	M349T15_2	87.1	6.45	64.26	2.49E-14	349.20	14.57	80	DOWN
21	2’‘-o-galloylisovitexin	M567T12	84.4	−6.40	−31.05	5.80E-07	567.11	11.96	76	DOWN
22	Indoleacrylic acid	M188T5	77.6	−6.28	−25.52	1.71E-06	188.07	5.28	61	DOWN
23	Hydroxyphenyl)naphthalic anhydride	M308T1	68.8	−6.11	−27.67	1.14E-06	308.09	1.00	35	DOWN
24	Anandamide (18:3, n-6)	M344T7	61.8	−5.95	−23.92	2.30E-06	344.26	6.87	90	DOWN
25	D-ribonolactone	M166T3	59.5	−5.90	−56.19	2.79E-08	166.07	2.71	84	DOWN
26	Heneicosanedioic acid	M379T23	47.5	−5.57	−44.06	4.07E-08	379.28	22.95	28	DOWN
27	Pantoprazole	M447T10	40.4	−5.34	−74.11	7.31E-09	447.09	9.66	2	DOWN
28	Sericetin	M443T1	35.7	−5.16	−46.98	7.21E-08	443.13	0.93	17	DOWN
29	Clidinium	M317T22	21.1	−4.40	−23.85	1.51E-06	317.18	22.37	40	DOWN
30	Anandamide (20:l, n-9)	M354T21	17.9	−4.16	−20.60	4.82E-06	354.34	20.63	11	DOWN
31	Quercetin 3-(2’‘,3’‘,4’‘-triacetylgalactoside)	M629T12	7.6	−2.92	−26.28	5.28E-08	629.09	11.97	76	DOWN
32	Catechin 5,3’-di-O-beta-D-glucopyranoside	M653T13	6.9	−2.79	−17.06	1.01E-08	653.15	12.98	161	DOWN
33	3e,13z-octadecadienal	M328T9	6.0	−2.58	−57.77	2.87E-10	328.26	8.88	48	DOWN
34	Caftaric acid	M335T5	5.9	−2.55	−11.50	4.55E-05	335.04	4.95	196	DOWN
35	Epicatechin monogallate	M443T2	3.6	−1.86	−9.50	2.51E-05	443.10	2.46	137	DOWN
36	Hexazinone	M270T5	2.2	−1.11	−11.93	7.20E-06	270.19	5.43	87	UP
37	Arginyl-proline	M254T3	2.1	−1.06	−17.78	1.49E-06	254.16	2.53	67	UP
38	Aminosalicylate sodium anhydrous	M176T7	1.9	−0.96	−10.77	6.36E-06	176.03	7.06	30	UP
39	Dihydrorobinetin	M327T3	1.8	−0.82	−32.31	2.65E-07	327.05	2.61	70	UP
40	Phlorin	M311T3	1.6	−0.65	−10.25	8.95E-06	311.07	2.60	70	UP

Compounds were selected based on fold change > 1.5.

The annotation results from METLIN revealed a wider diversity of compound classes compared to SIRIUS. While both platforms detected similar classes, METLIN additionally annotated compounds such as flavonoids, saponins, and organic acids. Notably, two alkaloids, actinonin and indole acrylate, were consistently identified by both SIRIUS and METLIN. Of particular interest, actinonin has been reported as a potent antibacterial alkaloid (Table 6) [42], highlighting the pharmacological relevance of the annotated metabolites.

**Table 6:** Compounds, targets and biological activities prediction using the BindingDB and Therapeutic Target Database.

Compound name	Target proteins	Drug structure similarity	Tanimoto Similarity	Type of Similarity	Therapeutic class	Bioactivity
Actinonin	Aminopeptidase N, Interstital collagenase, Neutrophil collagenase, Peptidase deformylase 1A, Peptidase deformylase, Stromelysis	Actinonin	1.0	High	Anti-bacterial agent	Staphylococcus Peptide deformylase (Stap-coc def)
Pacitron	5-hydroxytryptamine receptor 1A, 2A, 2B, 2C, 3A/3B, 4,7, Aceylcholestinerase, Indoleamine 2,3-dioxygenase	L-tryptophan	1.0	High	Anti-depressants agent	Indoleamine 2,3-dioxygenase 1 (IDO1)
1-butyl-3-[4-[2-hydroxy-3-(propan-2-ylamino)propoxy]phenyl]urea	Beta-1 adrenergic receptor, Beta-2 adrenergic receptor	Practolol	0.936	High	Anti-arrhythmic agent	Adrenergic receptor beta-1 (ADRB1)
Indol-acrylate	Trp operon receptor	Tryptamine	0.873	High	–	5-HT 2A receptor (HTR2A), Monoamine oxidase type A (MAO-A), Monoamine oxidase type B (MAO-B)
(2S)-1-[(2S)-2-[[(2S,3S)-2-amino-3-methylpentanoyl]amino]-5-(diaminomethylideneamino)pentanoyl]pyrrolidine-2-carboxylic acid	Complement factor B, Neuropilin-1, Non-structural protein 4A, Proprotein convertase subtilsin/kexin type 6	H-KPPR-OH	0.857	High	–	Neuropilin-1 (NRP1)
2-[4-(4-methoxyphenyl)-2,2-dimethyloxan-4-yl]ethyl-[(4-methoxyphenyl)methyl]azanium	Mu-type opioid receptor	1-(2-(3-methoxyphenyl)-1-phenylethyl)piperazine	0.851	High	–	Norepinephrine transporter (NET), Serotonin transporter (SERT)

### Multivariate analysis of *S. schimperi* untreated and treated extracts

**Fig 3:**
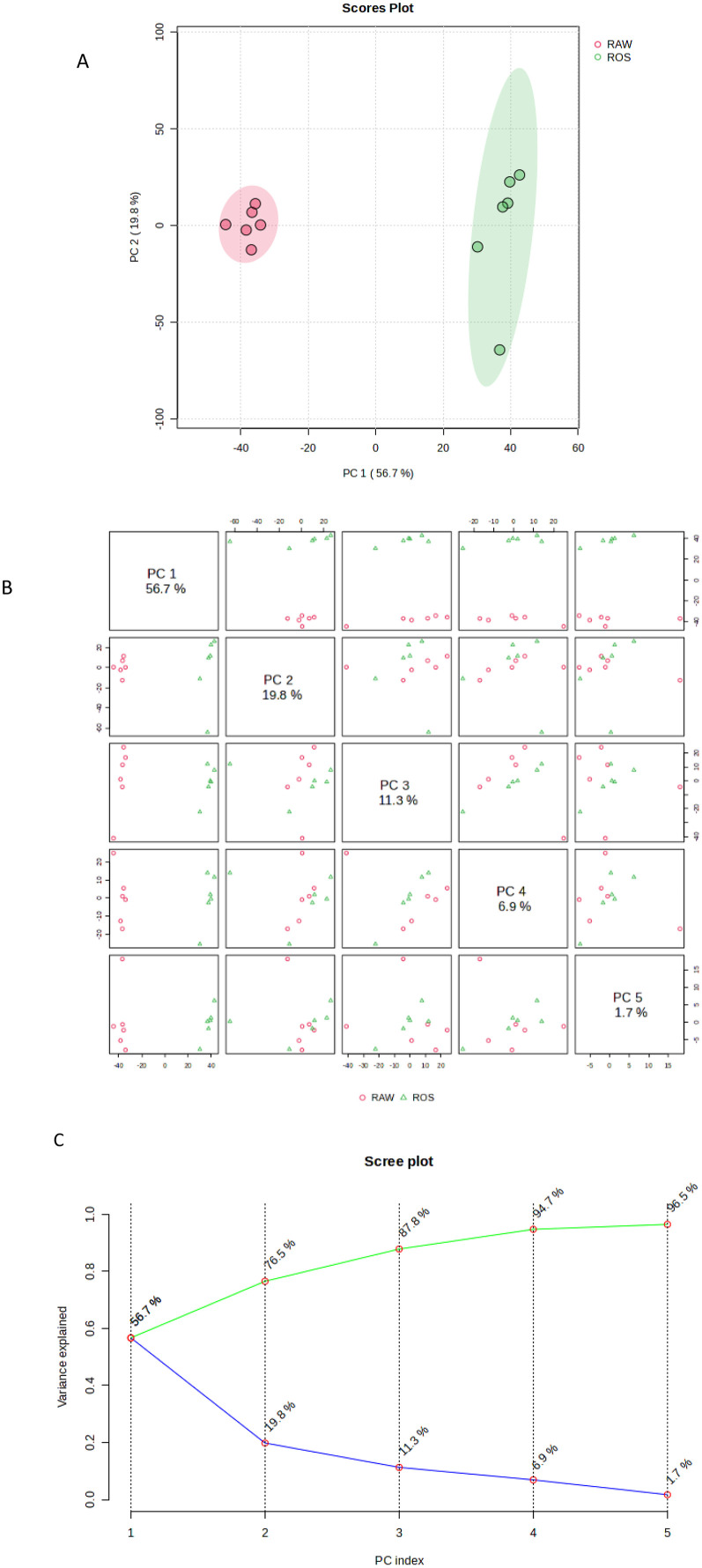
A. Multivariate data analyses of the seed extracts of the *S. schimperi* raw (untreated) (RAW) and roasted (treated) (ROS) showing their principal component analysis (PCA) score plot. B. Multivariate data analyses of the seed extracts of the *S. schimperi* raw (untreated) (RAW) and roasted (treated) (ROS) showing their paired score. C. Multivariate data analyses of the seed extracts of the *S. schimperi* raw (untreated) (RAW) and roasted (treated) (ROS) showing their Scree plot variance explained.

The principal component analysis (PCA) score plot showed distinct groupings between the seed extracts of the *S. schimperi* raw (untreated) (RAW) and roasted (treated) (ROS) (Fig 3A). The five principal components (PC) used in generating the PCA gave a total explained variance of 96.5% (Fig 3B,C). From these five PCs, the first two PCs [i.e., PC1 (56.7%) and PC2 (19.8%)] captured the most information from the given datasets (Fig 3B,C), which led to a clear separation between the treated and the untreated extracts.

**Fig 4:**
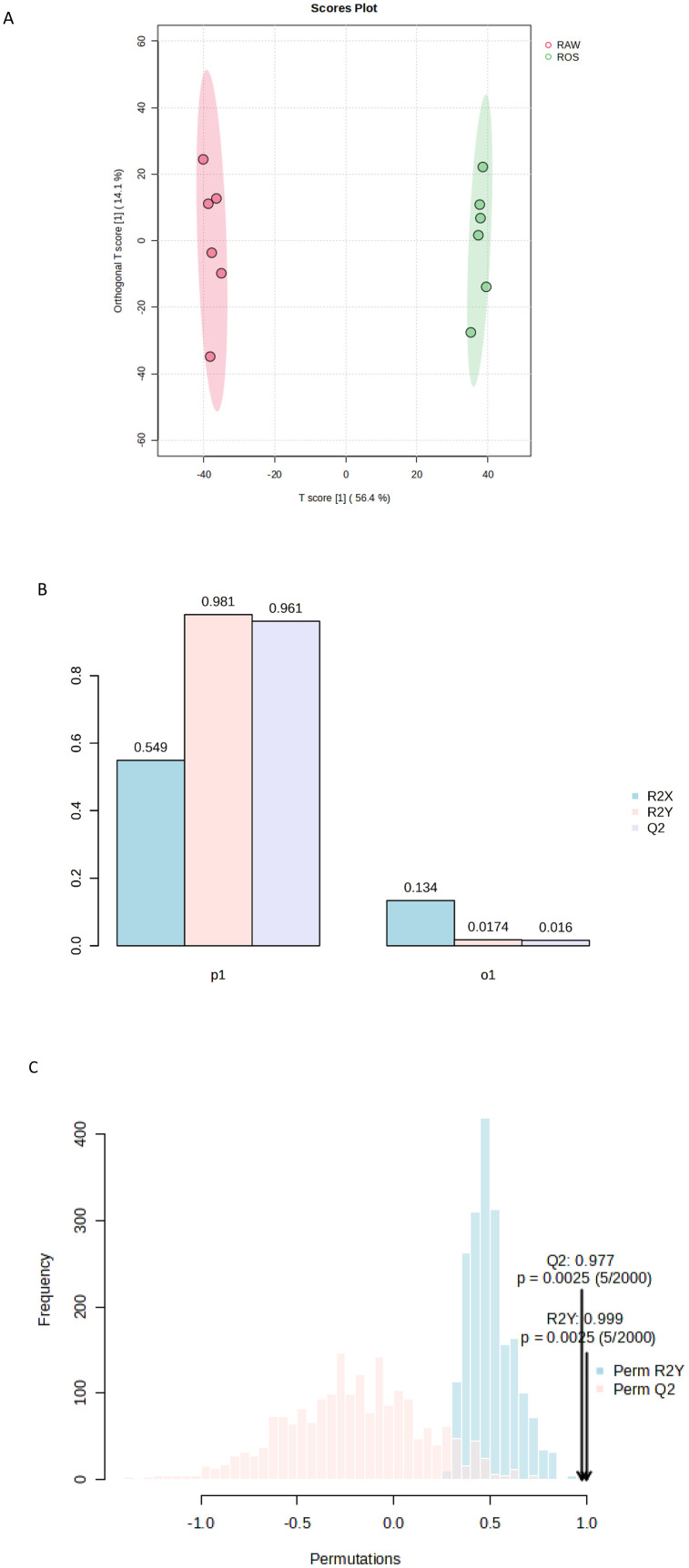
A. The multivariate data analyses of the seed extracts of the *S. schimperi* raw (untreated) (RAW) and roasted (treated) (ROS) showing their orthogonal partial least-squares discriminant analysis (OPLS-DA) classification using different number of components. B. The multivariate data analyses of the seed extracts of the *S. schimperi* raw (untreated) (RAW) and roasted (treated) (ROS) showing their cross-validation test. C. The multivariate data analyses of the seed extracts of the *S. schimperi* raw (untreated) (RAW) and roasted (treated) (ROS) showing their permutation test.

To further explain the groupings in the PCA, an orthogonal partial least-squares discriminant analysis (OPLS-DA) was performed (Fig 4). The score plot had a T score of 56.4%, which indicates that 56.4% of the variance between untreated and treated was explained (Fig 4A). The OPLS-DA score plot [Q^2^ = 0.977, R^2^Y = 0.999 (p < 0.01)] (Fig 4B,C) used to categorize the detected secondary metabolites as either from untreated or treated samples, effectively distinguishing between the two groups (Fig 4A). Authors have demonstrated the score plot to discriminated the secondary metabolites of mycelia of *Ganoderma boninense* into monokaryon and dikaryon [43]. Furthermore, the plot also showed an orthogonal T score of 14.1% indicating that 14.1% of the variations do not contribute to the separation of the groups, which reduced the “noise” in the model, thereby improving the accuracy of group separation (Fig 4A). The group classification via PCA and OPLS-DA is indicative of the distinction between the metabolite profile of untreated (RAW) and treated (ROS) of *S. schimperi* seed extracts.

**Fig 5:**
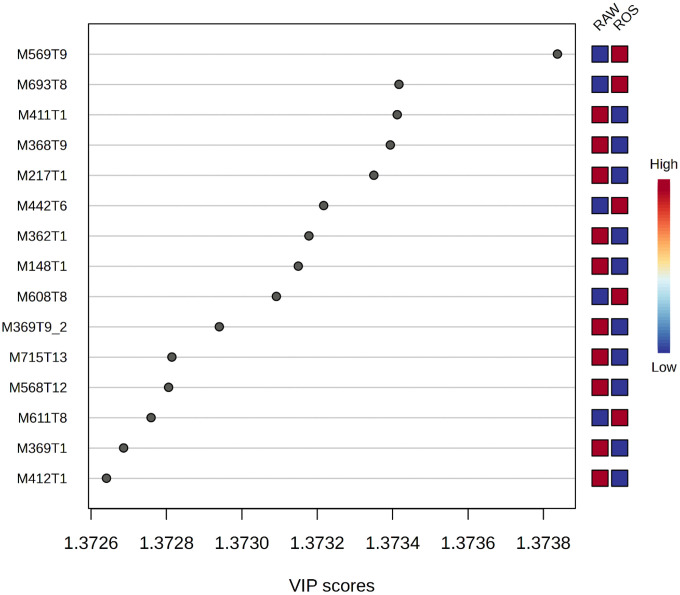
Volcano plots analysis of top 15 up-regulated and down-regulated metabolites of the untreated and treated extracts of *S. schimperi* ranked. Key: M365T1: Analyte, M453T8: Serine, M534T1: Analyte, M369T1: Miraxanthin-V, M474T1: Analyte, M399T7: Analytes, M400T7: Analyte, M188T3: L-Phenylalanine, M339T5: Phosphophosphinate, M217T21: 7,10-hexadecadienoic acid; M569T9: isoleucine, M704T9: coumaroylspermin, M222T7: (6E)-3,7-Dimethyl-6-octene-1,2,3,8-tetrol, M400T6: analyte, M432T3: tryptophan.

**Fig 6:**
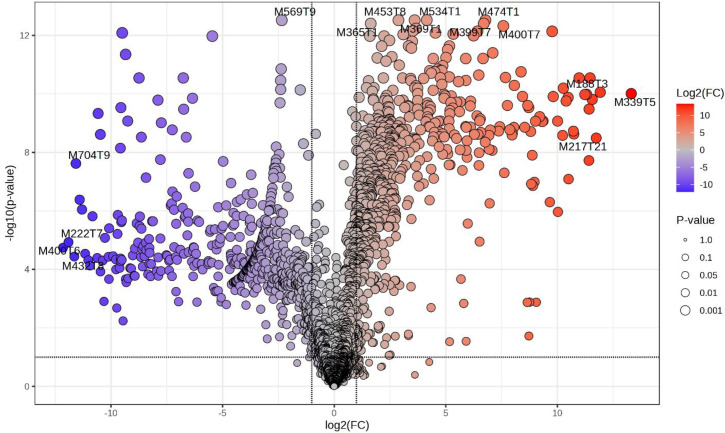
Distribution patterns of *untreated* and *treated* extracts of *S. schimperi* compounds. The scatter plot of VIP coordinates showing top 15 significant compounds (VIP > 1.2). Key: M569T9: isoleucine, M693T8: 3,5,3’-Triiodothyronine, M411T1: analyte, M368T9: himbacine, M217T1: metameconine, M442T6: L-aspartate, M362T1: funtumine, M148T1: glutamate, M608T8: glutamine, M369T9_2: analyte, M715T13: patuletin 3-(6’‘-(E)-feruloylglucoside, M568T12: analyte, M611T8: metrizoic acid, M369T1: octadecanedioic acid and M412T1: (S)-Autumnaline.

Univariate analysis was used where the volcano plot was made using fold change (FC) values (>2.0) and false discovery rate (FDR) corrected p-values (q-values) < 0.05, as the selection criteria for identifying the degree of metabolic molecule difference, as shown in Fig 4. A total of 1535 metabolites were not significant, while 572 were downregulated and 649 were upregulated (Fig 4). Among the 15 metabolites (Fig 5) with a VIP greater than 1.2, include M569T9 (isoleucine), M693T8 (3,5,3’-Triiodothyronine), M411T1 (analyte), M368T9 (himbacine), M217T1 (metameconine), M442T6 (L-aspartate), M362T1 (funtumine), M148T1 (glutamate), M608T8 (glutamine), M369T9_2 (analyte), M715T13 (patuletin 3-(6’‘-(E)-feruloylglucoside), M568T12 (analyte), M611T8 (metrizoic acid), M369T1 (octadecanedioic acid) and M412T1 ((S)-Autumnaline). Isoleucine, which was identified as a significant compound in the analyzed extracts, is one of the branched-chain amino acids that is an essential building block of protein synthesis in living organisms [44]. In this study among other compounds (3,5,3’-Triiodothyronine, L-aspartate, glutamine and metrizoic acid) it was also found to be more abundant in the roasted than in raw of *S. schimperi*. The compounds that were more abundant in the raw seeds of *S. schimperi* are himbacine, metameconine, funtumine, glutamate, patuletin 3-(6’‘-(E)-feruloylglucoside, octadecanedioic acid and (S)-Autumnaline. The top 15 downregulated (Fig 6) include M569T9: isoleucine, M704T9 (coumaroylspermin), M222T7 ((6E)-3,7-Dimethyl-6-octene-1,2,3,8-tetrol), M400T6 (analyte) and M432T3 (tryptophan) while the upregulated include M365T1 (Analytes), M453T8 (Serine), M534T1 (Analytes), M369T1 (Miraxanthin-V), M474T1 (Analyte), M399T7 (Analyte), M400T7 (Analyte), M188T3 (L-Phenylalanine), M339T5 (Phosphophosphinate) and M217T21 (7,10-hexadecadienoic acid). The downregulation of isoleucine observed in the roasted *S. schimperi* seed indicates that the raw seeds of the plant have more nutritional value than the roasted.

## Conclusion

This study revealed that *S. schimperi* seeds contain significant amounts of various chemical compounds, including flavonoids, saponins, alkaloids, small peptides, polyamines, amino acids, and organic acids. Notably, two alkaloids which are actinonin and indole acrylate, and the amino acid isoleucine were identified using SIRIUS and METLIN. Isoleucine, an essential amino acid, is known for its high nutritional value, while actinonin has been demonstrated to possess potent antibacterial properties. These findings have highlighted the potential value of *S. schimperi*, and further research into its toxicity and antimicrobial activities is recommended to fully harness its benefits.

## Supporting information

S1 Data PLos One Supplementary File. (XLSX)
